# Use and subjective experience of the impact of motor-assisted movement exercisers in people with amyotrophic lateral sclerosis: a multicenter observational study

**DOI:** 10.1038/s41598-022-13761-6

**Published:** 2022-06-10

**Authors:** André Maier, Marcel Gaudlitz, Torsten Grehl, Ute Weyen, Robert Steinbach, Julian Grosskreutz, Annekathrin Rödiger, Jan Christoph Koch, Teresa Lengenfeld, Patrick Weydt, René Günther, Joachim Wolf, Petra Baum, Moritz Metelmann, Johannes Dorst, Albert C. Ludolph, Dagmar Kettemann, Jenny Norden, Ruhan Yasemin Koc, Bertram Walter, Barbara Hildebrandt, Christoph Münch, Thomas Meyer, Susanne Spittel

**Affiliations:** 1 Outpatient Center for ALS and Other Motor Neuron Diseases, Charité—Universitätsmedizin Berlin, Corporate Member of Freie Universität Berlin, Humboldt-Universität Zu Berlin and Berlin Institute of Health, Augustenburger Platz 1, 13353 Berlin, Germany; 2 Ambulanzpartner Soziotechnologie APST GmbH, Berlin, Germany; 3 Department of Neurology, Alfried Krupp Krankenhaus, Center for ALS and Other Motor Neuron Disorders, Essen, Germany; 4 Center for ALS and Other Motor Neuron Disorders, Berufsgenossenschaftliches Universitätsklinikum Bergmannsheil, Bochum, Germany; 5 Hans Berger Department of Neurology, Jena University Hospital, Jena, Germany; 6 Precision Neurology, University of Lübeck, Lübeck, Germany; 7 Department of Neurology, Universitätsmedizin Göttingen, Göttingen, Germany; 8 Universitätsklinikum Bonn—Klinik Für Neurodegenerative Erkrankungen, Bonn, Germany; 9 Department of Neurology, Technische Universität Dresden, Dresden, Deutschland; 10 Research Site Dresden, German Centre for Neurodegenerative Diseases (DZNE), Dresden, Germany; 11 Department of Neurology, Diakonissenkrankenhaus Mannheim, Mannheim, Germany; 12 Department of Neurology, Universitätsklinikum Leipzig, Leipzig, Germany; 13 Department of Neurology, University of Ulm, Ulm, Germany; 14 German Center for Neurodegenerative Diseases (DZNE), Research Site Ulm, Ulm, Germany

**Keywords:** Neurology, Neurological disorders

## Abstract

Motor-assisted movement exercisers (MME) are devices that assist with physical therapy in domestic settings for people living with ALS. This observational cross-sectional study assesses the subjective experience of the therapy and analyzes users' likelihood of recommending treatment with MME. The study was implemented in ten ALS centers between February 2019 and October 2020, and was coordinated by the research platform Ambulanzpartner. Participants assessed symptom severity, documented frequency of MME use and rated the subjective benefits of therapy on a numerical scale (NRS, 0 to 10 points, with 10 being the highest). The Net Promotor Score (NPS) determined the likelihood of a participant recommending MME. Data for 144 participants were analyzed. Weekly MME use ranged from 1 to 4 times for 41% of participants, 5 to 7 times for 42%, and over 7 times for 17%. Particularly positive results were recorded in the following domains: amplification of a sense of achievement (67%), diminution of the feeling of having rigid limbs (63%), diminution of the feeling of being immobile (61%), improvement of general wellbeing (55%) and reduction of muscle stiffness (52%). Participants with more pronounced self-reported muscle weakness were more likely to note a beneficial effect on the preservation and improvement of muscle strength during MME treatment (p < 0.05). Overall, the NPS for MME was high (+ 61). High-frequency MME-assisted treatment (defined as a minimum of five sessions a week) was administered in the majority of participants (59%) in addition to physical therapy. Most patients reported having achieved their individual therapeutic objectives, as evidenced by a high level of satisfaction with MME therapy. The results bolster the justification for extended MME treatment as part of a holistic approach to ALS care.

## Introduction

Amyotrophic lateral sclerosis (ALS) is a fatal neurodegenerative disease that affects motor neurons, causing muscular atrophy and changes in muscle strength and tone. The most common disease presentation is limb onset, which is found in about two-thirds of people living with ALS. Almost all of these people living with ALS experience muscle weakness and stiffness of the arms and legs as the disease progresses^1^. While ALS is an inevitably progressive degenerative disease, the provision of assistive devices^2^ and physiotherapy are recommended and well-established therapeutic measures^3^. Given the variability of ALS progression, there are no uniform therapeutic regimens. Rather, there are a variety of individualized therapeutic approaches, which focus on strengthening and stretching muscles, regulating muscle tone, pain management, and preventing falls^4^. Multiple trials recommend regular moderate muscle strengthening and aerobic exercises at submaximal effort^3^. In a recently published meta-analysis, the individual effects of aerobic, resistance, and combined exercise training in patients with ALS remained unclear, although a subgroup analysis indicated a statistical significant effect in favor of combined training for upper and lower body strength^5^. Statistical differences attributable to physical therapy were evident in terms of maximizing oxygen uptake, improving quality of life, and maintaining functional abilities at the end of the therapeutic periods and six months thereafter. However, further high-quality studies with larger sample sizes are still needed. Given that people with ALS are at greater risk of infection, particularly when respiratory functions are compromised, the COVID-19 pandemic has highlighted the importance of a robust immune response. There is strong evidence that physical activity reduces mortality and improves disease outcome for people experiencing a symptomatic COVID-19 infection^6^.In addition to the positive effects mentioned, preclinical studies have demonstrated a correlation between physical activity and neuroprotection and prolonged motor neuron survival^7^.

The benefits of a regular mobility routine in combination with low to moderate intensity cycling exercises have been demonstrated in patients with ALS^8^. As motor-assisted movement exercisers (MME) have proven beneficial for treating other neurological diseases^9–11^, they have also been integrated into standard ALS care. Training options avaibale under MME include active (muscle powered), passive (motor powered) and assistive (muscle and motor powered) training for both arms and legs. The perceived benefits are a strengthening of muscle groups, reduction of spasticity, and support of the cardiovascular system^9,10^. In addition, MME treatment mobilizes the entire musculoskeletal system, including joints and tendons, while eliciting sensory inputs, which in turn enhance sensorimotor feedback^12,13^.

In Germany, people with ALS may be provided with assistive devices and technology, including MME, which can be used at home to complement physiotherapy. While this is part of standard care and in line with the current guidelines^14^, the provision of prescription exercise devices is dependent upon a patient’s individual medical situation.

To date, there have been no systematic studies on the utilization and provision of MME in ALS. As we are convinced that technology-assisted therapy will grow in importance, that exercise therapy for ALS patients is deserving of further research, and that the perspective of patients is essential to achieving these aims, we initiated this study to investigate patient satisfaction with MME by examining a cohort that was significantly larger than those of any previous related study.

The aim of this observational study was to identify the provision and utilization of MME in ALS in Germany, including patients’ attitudes towards the treatment. To do this, we established the following hypotheses: (I) There is a correlation between the frequency of MME use and self-reported symptoms (muscle stiffness and muscle strength). (II) The higher the frequency of use, the more participants report benefits in their self-assessments. (III) There is a correlation between patient recommendation of MME and the frequency of use or other clinical or epidemiological data.

## Materials and methods

### Study design

A multicenter cross-sectional study was conducted across 10 specialized ALS treatment centers in Germany between February 2019 and January 2021. This study was conducted in accordance with STROBE criteria^15^.

### Participants

Subjects who met the following criteria were included in this study: possible, probable or definitive diagnosis of ALS according to the revised El Escorial criteria^16^, medical indication for domestic use of an MME, provision of an MME by the case management network Ambulanzpartner Soziotechnologie (APST)^2,17–19^ and provision of informed consent to participate in this study. Participants with other severe life-limiting diseases or with clinically significant cognitive impairment were not eligible for this study.

### Ethical approval and consent to participate

The study was conducted in accordance with the relevant guidelines and regulations approved by the Medical Ethics Committee of Charité–Universitätsmedizin Berlin, Germany, under code EA1/219/15. Subjects were supplied with information about the study both verbally and in writing. Informed consent was obtained from all subjects.

### Setting

#### Case management

Prior to the study, all 10 contributing ALS centers already belonged to the APST multicenter case management network, in which it is standard practice for ALS-trained neurologists to medically indicate that MME therapy be embedded within ALS care. After APST case managers notify providers of a patient’s need for this device, participants are granted a four-week trial period, after which an individualized therapy regime is determined in close consultation with ALS-trained neurologists, physical therapists and assistive device providers. In Germany, the outcome of the trial period determines whether or not the costs incurred will ultimately be absorbed by the patient’s health insurance provider. Due to the non-interventional design of this study, a general recommendation for MME use was issued, although patients were not given specific exercise routines to follow.

#### Assessment and data capture

Case report forms were issued to neurologists, study coordinators and study assistants for the purpose of assessing clinical and demographic data. Print and online questionnaires were issued and structured interviews were conducted via the digital research platform APST for the purpose of assessing patient-reported outcomes.

### Variables

#### Demographic and clinical data

The demographic and clinical data collected included gender, age at symptom onset, disease duration, type of ALS, and a score derived from the self-administered ALS Functional Rating Scale-Revised (ALSFRS-R)^20^. The ALSFRS-R is a clinically validated and widely used diagnostic instrument that assesses the fine and gross motor functions of the arms and legs, bulbar functioning and ventilation. It comprises 12 short questions with 5 anchor points (0–4) as response options. The scale spans 0 to 48 points in total, with the bottom end of the scale reflecting low functionality and more pronounced disease severity. A monthly decline in ALSFRS-R points, or delta ALSFRS-R, is indicative of the rate of deterioration and has prognostic significance^21^.

#### Self-assessment of muscle stiffness and muscle weakness

We intentionally used the terms "muscle stiffness" and "muscle weakness" to describe ALS symptoms, as this reflects how individual participants evaluated and perceived their symptoms. The ratings of participants did not necessarily correspond to the clinical picture of patient spasticity and paresis. Arms and legs were assessed separately on a numeric rating scale (NRS), with a score of 0 signifying no stiffness/weakness and a score of 10 signifying the most pronounced stiffness/weakness.

#### MME use

The frequency of MME use was delineated as follows: once or twice a week, 3 to 4 times a week, 5 to 7 times a week, 8 to 10 times a week, and more than 10 times a week.

#### Recommending the MME

The Net Promoter Score (NPS) was used to evaluate participants’ attitudes towards the MME, and their overall rates of recommendation^22,23^. Originally a tool created for customer relations management, the NPS has since been applied as an instrument for evaluation in various clinical settings^19,24–26^. For this clinical study, we calculated NPS scores in response to the question: “How likely is it that you would recommend the MME to another friend or patient who is affected with ALS?”.

Based on an 11-point Likert scale from 0 (recommendation absolutely unlikely) to 10 (recommendation highly likely), participants who gave the MME a 9 or 10 were considered "promoters" (likely to recommend), those who gave it 7 or 8 points were considered "indifferent," and responders who gave the MME 6 to 0 points were considered "detractors" (unlikely to recommend). Ultimately, the percentage of detractors was subtracted from the percentage of promoters to ascertain a final score. In general, a group with a positive NPS is regarded as supportive, and results over 50 are considered excellent^23^. To avoid categorization difficulties, it is also possible to omit the NPS calculus and merely report the average values corresponding to a recommendation^22^.

For the purposes of this study, we analyzed the NPS ratings of all participants, as well as of individual groups as determined according to gender, age, disease severity and frequency of use.

#### Domain-oriented participant experience with MME

To record the subjective impacts of MME on an individual under treatment, an expert team of ALS neurologists and scientists with expertise in ALS care and observational studies developed statements around nine items. The wording of these statements was crafted so as to reflect how a patient might perceive the changes that may occur during ALS. We deliberately avoided medical-scientific terms such as “spasticity,” “contractions” or “joint locking.” Rather, patients were questioned about “preservation of muscle strength,” “improvement of muscle strength,” “reduction of muscle stiffness,” “reduction of tendon shortening,” “diminution of the feeling of having rigid limbs,” “diminution of the feeling of being immobile,” “amplification of a sense of achievement,” “improvement of sleep quality” and “improvement of general wellbeing.” Patients were asked, “How do you rate the benefits you personally gained with the MME? Please specify for each domain.” Participants expressed their agreement or disagreement by giving each statement a rating between 0 (no impact) and 10 points (best possible impact) on a Likert scale.

### Statistical methods

Data was analyzed with IBM SPSS Statistics (Version 25.0). If individual data points of an item were missing, total scores reliant on those items were excluded from statistical analysis. Descriptive analyses were conducted to compare frequencies within the assessed parameters. Results were expressed as means (± SD) if distribution was normal, and as medians if numerical data was visualized or if distribution was non-Gaussian. Significant differences between the parameters and, respectively, subgroups of nominally scaled data were assessed by applying contingency tables and Chi-square test. Statistically significant differences of paired samples were analyzed by t-tests. The Wilcoxon test was employed to analyze the statistical power of ordinally-scaled data, while metric data were subjected to the t-test. Correlational analysis was performed with Spearman’s Rho because of the ordinal nature of the scales. For comparative purposes within the parameters of weakness and stiffness, a ratio was devised for each participant. To discern group differences within nonparametric data, the Mann–Whitney *U* test was performed on two independent samples, and the Kruskal–Wallis one-way analysis of variance of independent samples was conducted on three or more. Statistical significance was ascertained with a risk of error of up to 5% (p-value < 0.05).

## Results

### Sample characteristics

Within the observation period, 237 patients were identified for whom APST case management initiated the provision of MME, All of these were subsequently invited to participate in the study. Ultimately, the respective study centers enrolled 144 patients in total.

### Demographic data and clinical characteristics

The gender distribution (male:female) was 1.5:1, which is a common distribution across the general population of ALS patients^27,28^. The age of ALS onset was within the general range of prevalence in the total ALS population (59.9 years SD 11.5)^29^. At the time of initial MME use, the average disease course was 37.3 months (3.1 years). The survey was conducted after an average MME use time of 8.2 months (SD 10.9), at which point the degree of impairment was severe, averaging 27.2 ALSFRS-R points (SD 9.8), and the disease was progressing at an average rate of loss of approximately 0.7 points per month.

Self-assessment revealed that 83.7% of participants experienced muscle stiffness in both legs and arms, and almost all (97.1%) experienced weakness in the extremities at the time of the survey. Results showed that both muscle stiffness and weakness were more pronounced in the legs. Only 17% of all patients reported experiencing only one of these symptoms.

In total, muscle weakness was significantly higher than muscle stiffness (p < 0.001). Weakness correlates with disease severity, as measured by the ALSFRS-R (p < 0.001; correlation coefficient = 0.4) whereas no such correlation could be established with regard to muscle stiffness. Further information on demographic and clinical characteristics are presented in Table 1.

**Table 1:** Demographic and clinical characteristics of the cohort.

Characteristics	Classification	Total cohort, n = 144
Sex	Female, % (n)	36.8 (53)
	Male, % (n)	63.2 (91)
Age	At onset, years, mean (SD, R)	59.9 (11.5; 25.8–81.5)
	At time of MME† use, years, mean (SD, R)	62.1 (11.1; 29.6–82.8)
Disease duration	At time of MME† use, months, mean (SD, R)	37.3 (49.7; 3.0–513.0)
Disease progression	Mean (SD, R)	0.7 (0.6; 0.0–2.8)
ALS-FRS-R score (max. 48)	At time of survey, mean (SD, R)	27.2 (9.8, 1.0–40.0)
Presence of muscle stiffness	Total, yes, % (n)	83.7 (113)
	In lower extremities, % (n)	73.5 (97)
	In upper extremities, % (n)	69.6 (94)
Severity of muscle stiffness‡	Mean (SD, R)	3.7 (2.7, 0–9)
	In lower extremities, mean (SD, R)	4.0 (3.2; 0–10)
	In upper extremities, mean (SD, R)	3.4 (3.1; 0–10)
Presence of muscle weakness	Yes, % (n)	97.1 (133)
	In lower extremities, % (n)	94.1 (127)
	In upper extremities, % (n)	91.8 (123)
Severity of muscle weakness‡	Total, mean (SD, R)	4.6 (2.3; 0–10)
	In lower extremities, mean (SD, R)	4.8 (2.8; 0–10)
	In upper extremities, mean (SD, R)	4.3 (2.8; 0–10)

*n* number of participants, *SD* standard deviation, *R* range, *ALS-FRS-R* Amyotrophic Lateral Sclerosis Functional Rating Scale, revised, *MME* Motor-assisted movement exerciser.

^†^Disease duration at the time of the initial MME use.

^‡^Severity of muscle stiffness/weakness was assessed via a numeric rating scale (NRS) with 0 meaning no stiffness or weakness and 10 designating the most stiffness or weakness.

### Use of motor-assisted movement exerciser (MME)

**Figure 1:**
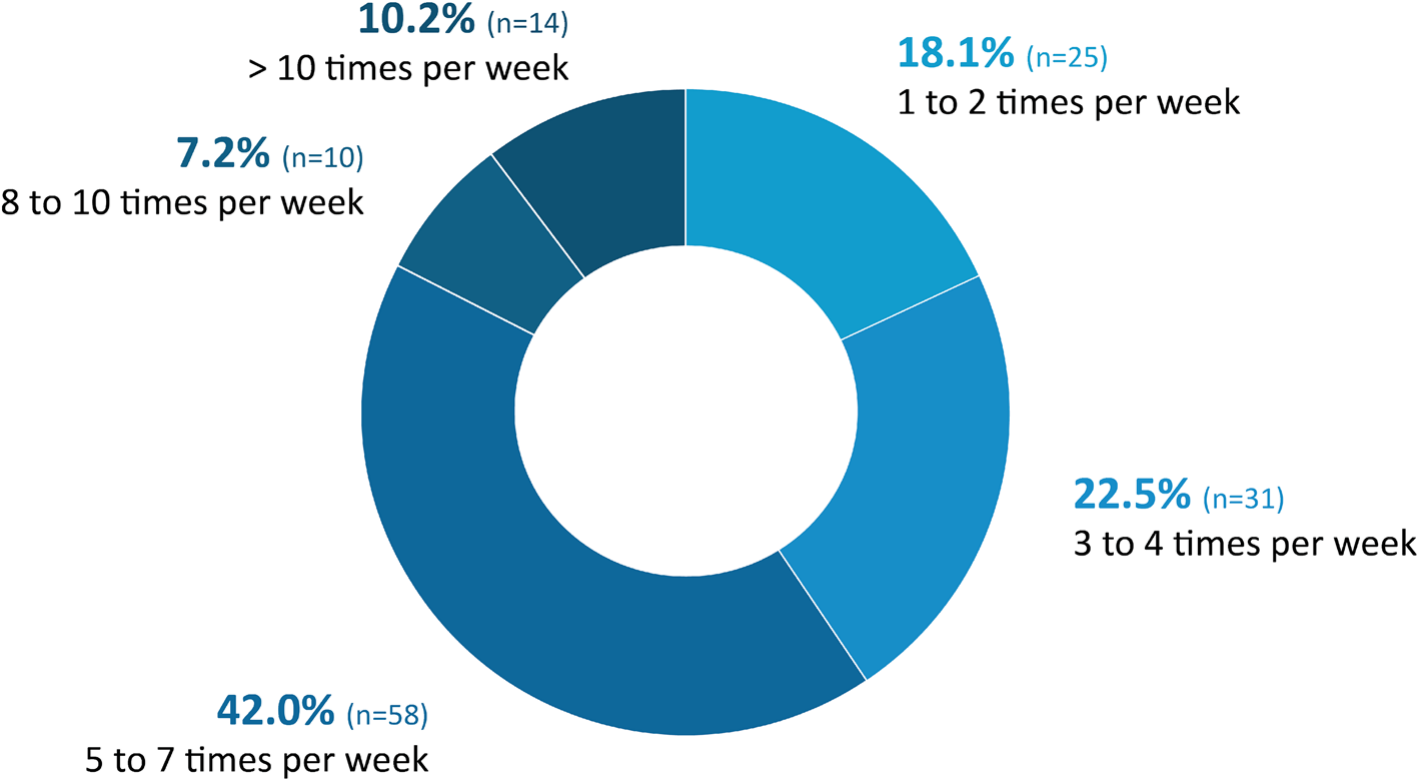
Use of motor-assisted movement exerciser (MME). MME use was broken down into the following categories: 1 to 2 times per week, 3 to 4 times per week, 5 to 7 times per week, 8 to 10 times per week, and more than 10 times per week. n = 138; *n* number of participants.

During the survey, the majority of participants (n = 138) exercised with MME at least 5 times a week (59.4%, n = 82). Additionally, 17.4% of participants surveyed (n = 24) used MME more than once a day on occasion (≥ 8 times per week; Fig. 1).

Only two participants (1.4%) had not yet started using the MME and another two participants (1.4%) were unable to use it anymore due to disease progression.

### MME recommendation

**Figure 2:**
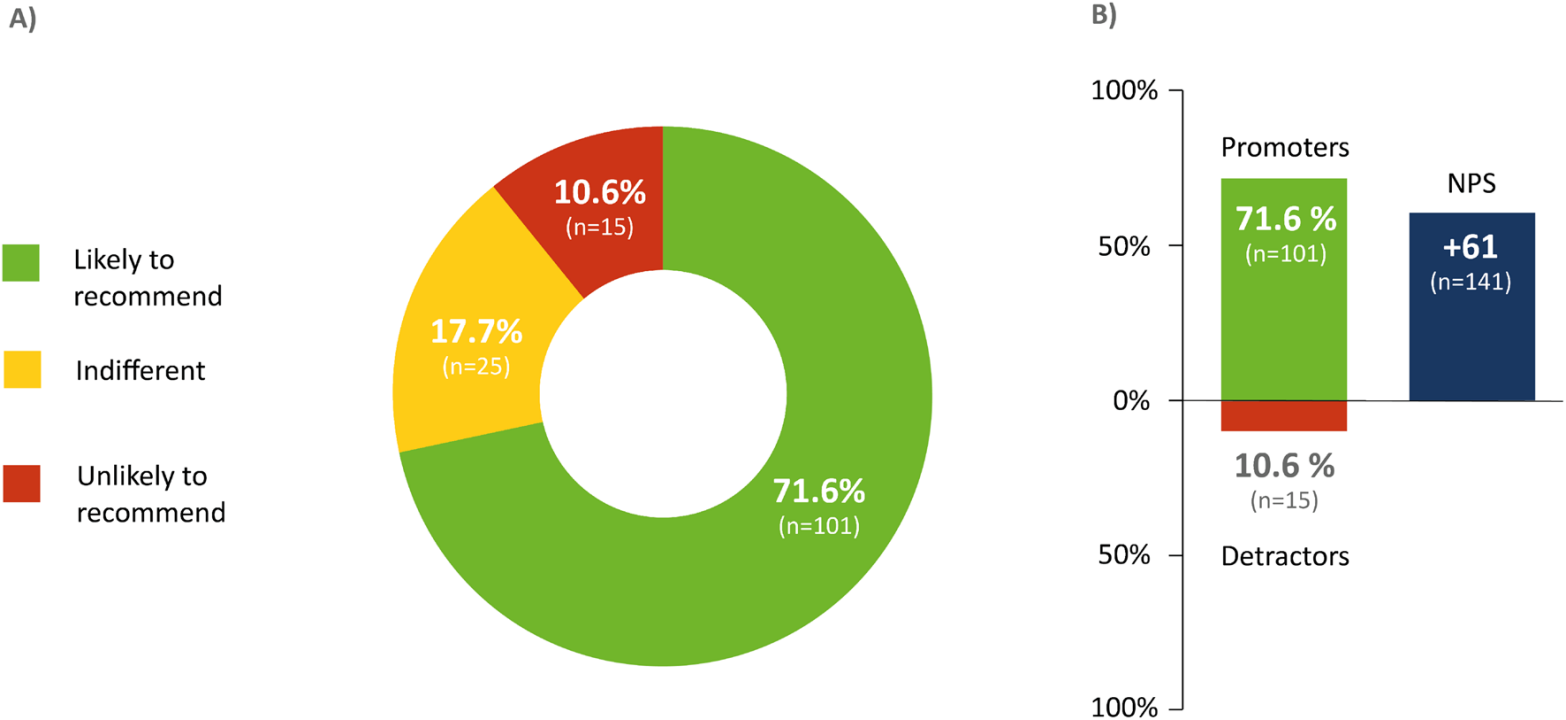
Likelihood of recommending a motor-assisted movement exerciser (MME). The NPS was used to assess participants’ likelihood of recommending the MME. The score was calculated based on responses to a single question: “How likely is it that you would recommend the MME to another friend or patient who is affected with ALS?” Answers were ranked between 0 (absolutely unlikely to recommend) and 10 (very likely to recommend). Participants who responded with a score of 9 or 10 were considered “promoters.” Those who gave the treatment a 7 or 8 were classified as “indifferent,” and participants whose rankings were between 0 and 6 were defined as “detractors” (**A**). The NPS was calculated by subtracting the percentage of detractors from the percentage of promoters (**B**). n = 141; n, number of participants.

71.6% of the responders (n = 101) strongly recommended MME (9 to 10 points). 17.7% (n = 25) were indifferent (7 to 8 points), and 10.6% (n = 15) did not issue a recommendation (0 to 6 points). The overall NPS was + 61 (Fig. 2).

**Figure 3:**
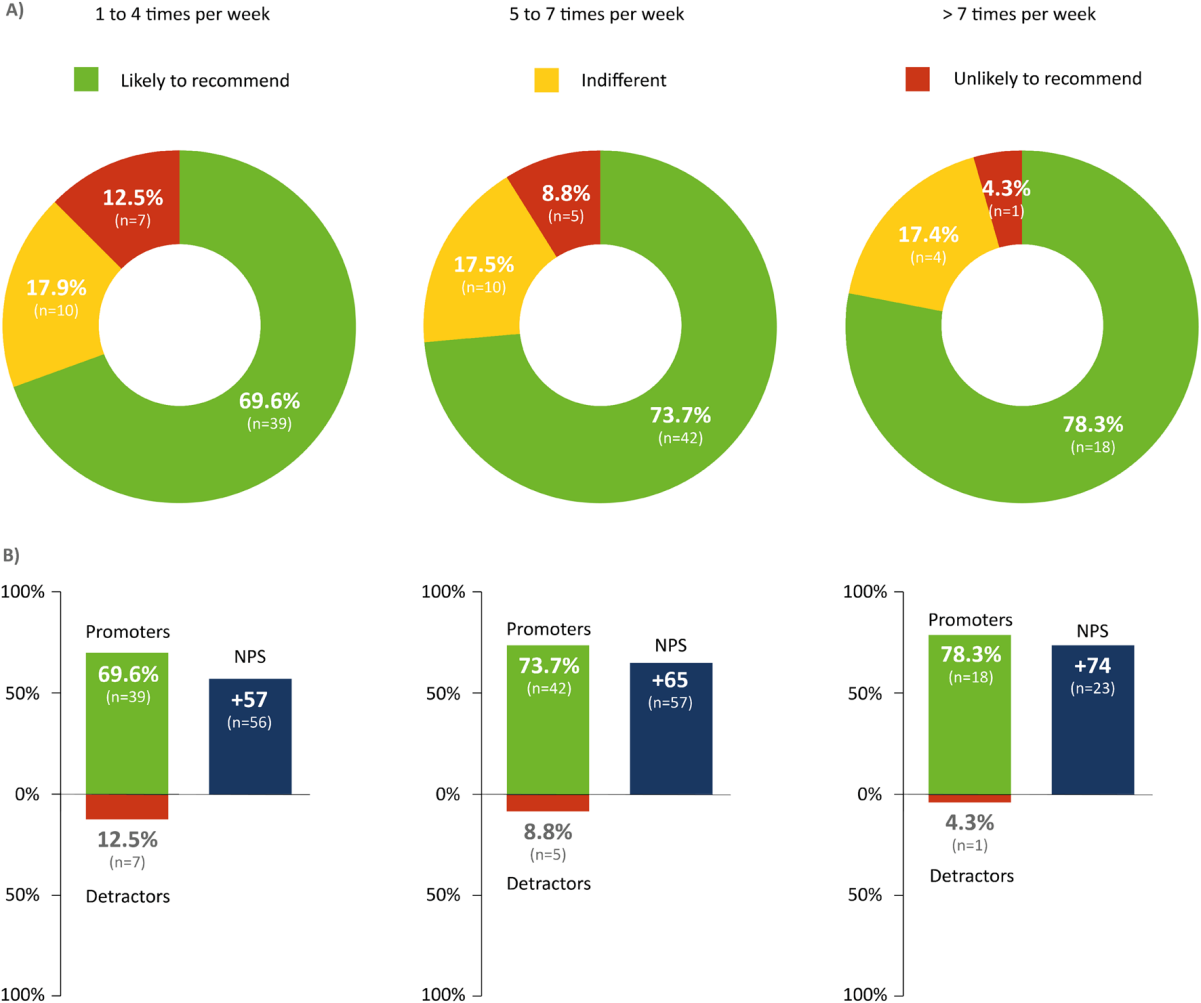
Willingness to recommend the motor-assisted movement exerciser (MME) with respect to frequency of MME use. The NPS was applied to assess participants’ likelihood of recommending the MME. This score was calculated based on responses to a single question: “How likely is it that you would recommend the MME to another friend or patient who is affected with ALS?” Answers were rated between 0 (absolutely unlikely to recommend) and 10 (very likely to recommend). Participants who responded with a score of 9 or 10 were considered “promoters.” Those who scored the treatment between 7 and 8 were considered “indifferent,” and participants who responded with between 0 and 6 points were defined as “detractors” (**A**). The NPS was calculated by subtracting the percentage of detractors from the percentage of promoters (**B**). n = 136; *n* number of participants.

A further analysis of the frequency of MME use showed that participants who used the device at least 5 days a week (5 to 7 times a week) and several times a day (> 7 times a week; Fig. 3) on some days were more likely to recommend the exerciser than other study participants.

Interestingly, recommendation rates did not differ significantly among participants who reported either greater stiffness or greater weakness in the extremities. Women were more likely to recommend MME than men (NPS: 67 vs. 58, p = 0.019). Participants over 60 provided higher NPS ratings than younger responders, however, this finding was not of statistical significance (NPS: 65.5 vs. 54, p = 0.58). Disease severity, as measured by the ALSFRS-R, did not have a significant impact on MME recommendation rates (ALSFRS-R groups subdivided by mean value: ≤ 27 vs. > 27: NPS 68 vs. 59; p = 0.531).

### Domain-oriented participant experience

**Figure 4:**
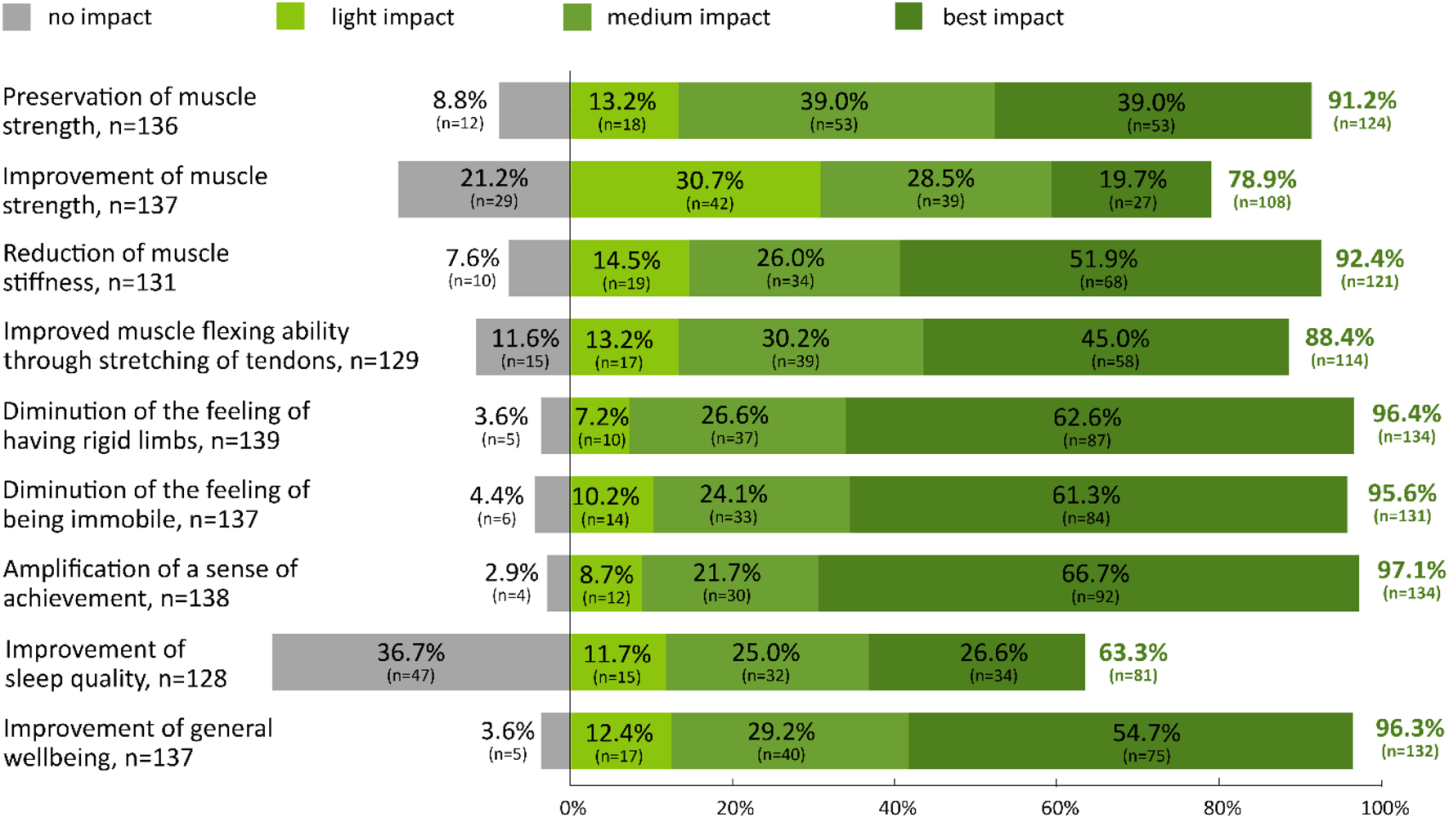
Domain-oriented participant experiences of the MME. Participant experiences of the MME as analyzed by domain were assessed on a 11-point Likert scale ranging from 0 (no impact) to 10 (best possible impact). The impacts were classified into four groups: no impact (0 points), light impact (1 to 3 points), medium impact (4 to 6 points), and most impact (7 to 10 points). MME, motor-assisted movement exerciser; n, number of participants.

The following percentages pertain to the highest impact on specific treatment areas as described by participants and as reflected by a score of 7 to 10 (Fig. 4). In descending order: amplification of a sense of achievement (67%), diminution of the feeling of having rigid limbs (63%), diminution of the feeling of being immobile (61%), improvement of general wellbeing (55%), reduction of muscle stiffness (52%), improved muscle flexing ability through stretching of tendons (45%), preservation of muscle strength (39%), improvement of sleep quality (27%), and improvement of muscle strength (20%).

**Figure 5:**
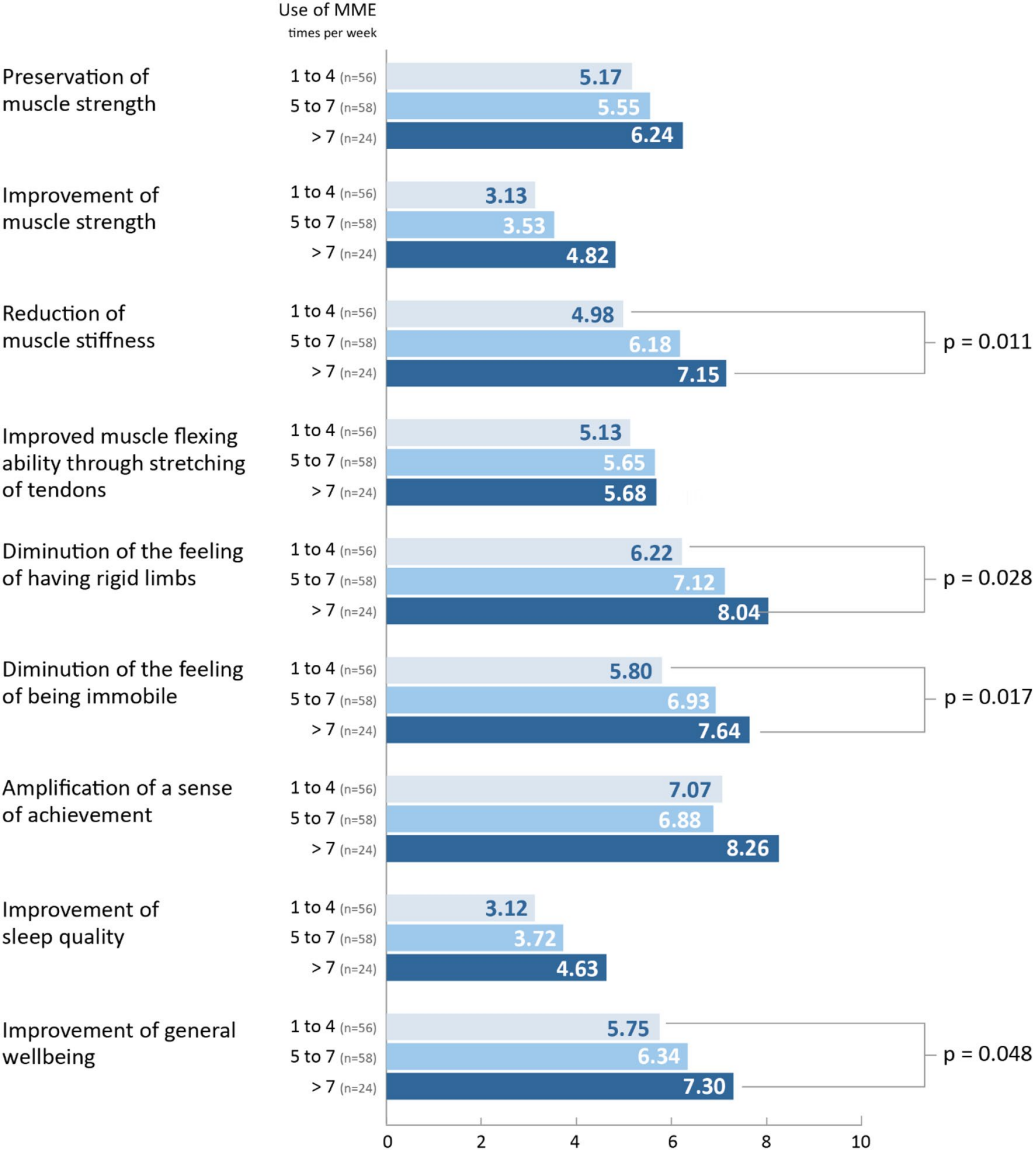
Domain-oriented participant experiences with respect to frequency of MME use. Domain-oriented participant experiences of MME were assessed on an 11-point Likert-scale ranging from 0 (no impact) to 10 (best possible impact). Different frequencies of MME use is depicted in the blue bars. Significant differences were assessed via the Kruskal–Wallis-Test. A p-value < 0.05 was considered significant. MME, motor-assisted movement exerciser; n, number of participants; n = 138.

There was a correlation between the reported overall benefit of MME treatment and frequency of MME use. This was highest among participants who conducted more than 7 exercise sessions a week. A significant correlation was found between frequency of use and the domains “reduction of muscle stiffness,” “diminution of the feeling of having rigid limbs,” “diminution of the feeling of being immobile,” and “improvement of general wellbeing” (Fig. 5).

Participants with severely stiff limbs did not express a preference for specific MME treatment target. Participants with higher self-rated muscle weakness were significantly more likely to see the beneficial effects of MME in terms of preserving and improving muscle strength (p < 0.05). Different age and gender groups reported similar experiences in all domains.

Although participants with greater weakness in the extremities gave higher ratings with regard to overall improvement of muscle strength via MME, this applied predominantly to patients in the earlier stages of the disease, as measured by the ALSFRS-R (p < 0.05).

## Discussion

### Sample selection, demographic and clinical characteristics

The motor-assisted movement exerciser (MME) facilitates device-assisted physical therapy in a home setting to treat motor symptoms of the extremities in people with ALS and other neurological diseases.

Focusing on 144 participants with a confirmed diagnosis of ALS, this is the largest study to date with the aim of investigating user satisfaction with MME treatment and the treatment’s therapeutic efficacy. With such a substantial number of participants, this study was able to reflect the general ALS population in terms of gender distribution, age at onset, and rate of disease progression. Furthermore, the multicenter study design reflects the broad base across which the digitally supported APST care network operates. Selection criteria for participants included medical indication for an MME and involvement in the care network, which are available primarily at specialized ALS centers.

Given that MME treatment was on average initiated 37 months after the onset of ALS, the mean disease duration following the treatment was notably long—especially considering average ALS survival time after symptom onset is 3–4^28,29^. This may explain the high level of disease severity at the time of the survey, i.e., 8 months after initial MME treatment. This, of course, raises the question of what the ideal point for issuing a MME prescription is, and when and how the provision process should be started. As determined by a previous study, provision failure rates are high (47%) and, with an average of 110 days, latency periods between medical indication for an assistive device and the device’s actual provision were remarkably high^2^. Several barriers were discovered to have prevented the early initiation of therapy, such as delays to issuing a medical indication, or a time-consuming, intensive, payer-side review of the indication prior to reimbursement. As one of the guiding objectives in the treatment of ALS is the preservation of functionality, exerciser-based treatment should be considered right at the initial onset of symptoms in affected extremities to counter the detrimental effects of the disease. Our clinical experience supports this assumption, but we will need to conduct further studies, preferably prospective in design, to provide conclusive clarification.

### Self-assessment of symptoms and domains

We would like to stress that the decision to rely on self-reporting for measuring symptom severity and prevalence was, by design, deliberate. Clinical experience shows that individuals who are affected with ALS but have no spasticity also report muscle stiffness, while the general presence of spasticity may also be perceived as weakness.

Overall, muscle weakness was reported to be more severe and more frequent than muscle stiffness (97% vs. 84%), and weakness was more pronounced in the legs than in the arms. 83% of participants reported the presence of both symptoms. Interestingly, muscle weakness correlated with both disease severity and progression rate, whereas no such correlation could be established for muscle stiffness. Although self-reporting on motor symptoms is naturally subjective, our observations demonstrate the differential significance of weakness and stiffness within disease progression.

### Characteristics of MME use and use frequency

The MME provides patients with high-frequency, motor-assisted physical therapy in the comfort of their own homes as an adjunct to physical therapy. It would be of interest to find out if access to standard physical therapy was more difficult to obtain for patients making frequent use of MME. Especially in regions or during periods when access to standard physiotherapy is limited (e.g. during the COVID-19 pandemic), regular MME sessions can compensate for a general lack of exercise.

In our study, MME use was typically extensive, as the majority of participants engaged in at least five weekly exercise sessions. There was no correlation between routine frequency and symptom severity or ALS disease progression as measured by the ALSFRS-R. Participants determined for themselves how often they would use MMEs. Each patient was given an individual recommendation for an MME by an attending therapist before the device was provided. Further adjustments were made over the course of treatment. Therapeutic recommendations were issued independently of our observational study, and were based on each individual user’s abilities and aims. At all times, participants were free to adapt the frequency and intensity of MME therapy to their specific conditions. Patients’ ultimate preferences surrounding the frequency of MME therapy may have impacted the assessment of the treatment’s overall benefit. Still, the sheer number of documented exercise sessions testifies to how well this device was received.

### Treatment recommendations and perception of MME therapy

Domain-based user assessments showed moderate benefits for the preservation of muscle strength. By comparison, participants rated MME more highly for its positive effects on stiffness. The domain of “stiffness” includes the criterion of ‘improved muscle flexing ability through stretching of tendons.” A substantially reduced ability to flex one’s muscles is a relevant problem in ALS, as it leads to contractures and can further limit a patient’s range of movement.

Participants most often reported that the sensation of being immobile and having rigid limbs was diminished by MME treatment, and that they enjoyed a sense of accomplishment and improved general wellbeing. The participants who reported their muscles feeling less stiff, a diminished sensation of having rigid limbs or feeling immobile, and feeling better in general, were those who used MME more often than other study participants. The likelihood of perceiving a positive correlation between MME treatment and the preservation and improvement of muscle strength was higher among participants in the earlier stages of the disease who rated their muscle weakness as more severe than average. In view of the fact that most participants strongly recommended MME, and women and older participants especially so, we have come to the conclusion that the treatment’s benefits are not particular to any specific group, but rather a highly individual matter within an ALS cohort. Subsequently, it makes little sense to prescribe MME treatment exclusively on the basis of either muscle stiffness or weakness, respectively.

## Limitations

The present study results relies on the analysis of participants’ subjective responses. Patient selection was based on individual indications for MME treatment, actual supply of devices, and willingness to participate in the study. While this is implicative of a selection bias and constitutes a limitation, the sample size of the study is such that the actual impacts of these factors are not all that strong. Certainly, a randomized trial that includes patients not receiving MME treatment could potentially produce stronger conclusions about certain parameters of treatment success. However, in light of our real-world limitations of our approach, that was not possible for this study.

With regard to the strength of results according to strengthening muscles and reducing spasticity or muscle stiffness, we must differentiate between evaluations performed by patients and those by distinguished medical professionals. Again, however, it was the purpose of this study to focus on the subjective experiences of treatment, i.e., on the fine interplay between device-based training and the personal experiences and opinions of patients, and their subsequent willingness to recommend the treatment. Such perceived benefits cannot always be captured by strictly objective measurement techniques.

Although definitive medical criteria for diagnosing ALS were applied in this study, the system used in this study within which symptoms were reported has not been validated**.** Therefore, we relied upon the subjective reports of patients, i.e., user perspectives, to assess the effectiveness of the treatment on specific symptoms. Further studies are needed to medically define and measure the effects of MME use on specific functionalities.

## Conclusion

This study demonstrates that patients report deriving high levels of benefit from MME treatment. Notwithstanding, the distinction between muscle weakness and stiffness remains equivocal. While positive effects are separately attributed to each cluster of symptoms; in reality, these categories cannot be regarded separately. One of the key characteristics of MME is its integration of active and passive exercises; that is, it allows for motor-assisted and passive limb movement as well as for active exercise. Due to the severity and progress of symptoms in ALS patients, most of the participants in this study would likely not have been able to pursue an active exercise regime only. In view of this fact, the MME gains both value and importance, and distinguishes itself from the various therapeutic and assistive devices currently available to patients. Motor-assisted movement exercisers have the capacity to play a crucial support role in the treatment of degenerative neurological diseases such as ALS.

In conclusion, the beneficial effects of MMEs are perceptible beyond a merely functional level. The findings of this study—namely, that patients undergoing MME treatment experienced enhanced wellbeing and the resolution of perceived inactivity—are consistent with the findings of a previous study^30^. Our study also suggests that MME treatment may have more positive psychological than physical benefits for people with ALS. However, it is also the case that in studies of progressive motor diseases, physical outcomes are especially difficult to demonstrate.

Although the MME is a medically indicated device that has received positive user feedback, it is not frequently supplied to patients. We can only speculate why this is the case. Further studies are needed to elucidate what precisely the obstacles are to early therapeutic implementation and to a more consistent provision process.

## Data Availability

The data that support the findings of this study are available from Ambulanzpartner Soziotechnologie APST GmbH. However, because this data was used under license, it is not publicly available. It can be obtained from the author Susanne Spittel (s.spittel@ambulanzpartner.de) upon reasonable request and with permission of Ambulanzpartner Soziotechnologie APST GmbH.

## Abbreviations

ALSFRS-R: ALS functional rating scale—revised
MME: Motor-assisted movement exerciser
NPS: Net promotor score
NRS: Numeric rating scale
SD: Standard deviation
